# Supplementary material for: Dynamic transcriptomic profiles of zebrafish gills in response to zinc supplementation
Source: BMC Genomics. 2010 Oct 11;11:553. doi: 10.1186/1471-2164-11-553 (PMC3091702; doi:10.1186/1471-2164-11-553)
Supplement: Additional file 2 — Interactive Direct Interaction Network representing the molecular interactions between zinc, copper, iron, calcium and proteins encoded by transcripts changed by zinc supplementation. Mini web-site containing index.html and hyperlinked pages in subdirectory describing a Direct Interaction Network automatically generated based on curated interactions contained within the proprietary PathwayArchitect database. Ovals represent proteins and the circles symbolize metal ions. Objects are coloured by their abundance in zebrafish at the time-point they were significantly different from the control is a scale from -4 fold (dark green) to +4 fold (dark red). Where significant differences were found at more than one time-point, the colour overlay shows expression at the first instance. Dark blue squares denote 'binding', and light blue squares 'expression'; green squares stand for 'regulation', green diamonds for 'metabolism', and green circles for 'promoter binding'. Arrow heads indicate directionality of the interaction where annotated. All nodes and edges can be further interrogated by selecting the relative area of the image. [file 1471-2164-11-553-S2.zip › PathwayArchitect Zn xs DIN/716541.html]

# REGULATION:

|  |  |
| --- | --- |
| Type | REGULATION |
| Effect | Positive |


---

|  |  |
| --- | --- |
| Score | 0 |


---

|  |  |
| --- | --- |
| Reference Count | 7 |


---

|  |  |
| --- | --- |
| Mechanism | Unknown |


---

|  |  |
| --- | --- |
| Reference:0 || Sentence | "The effect of fortification iron (reduced iron) on zinc absorption from a commercial vegetable-based weaning food was assessed in 11 9-mo-old infants." |
| PMID | 7572710 |
| Year | 1995 |
| Species | Human |
| Journal | Am J Clin Nutr |
| RefScore | 0 |
| Source | PArchNLP |
  |
|  |  |


---

|  |  |
| --- | --- |
 Reference:1 || Sentence | "Although it has been shown that IRA is associated with elevated plasma copper and depleted zinc levels in children, there are conflicting results on the effect of iron supplementation on the absorption of these elements." |
| PMID | 12907830 |
| Year | 2003 |
| Species | Human |
| Journal | Biol Trace Elem Res |
| RefScore | 3 |
| Source | PArchNLP |
  ||  |  |


---

|  |  |
| --- | --- |
 Reference:2 || Sentence | "This study reports the effect of paraquat (PQ) on concentrations of four elements (Cu, Fe, Mg, Zn) in lung, kidney, spleen, liver, and heart of male osteogenic disorder Shionogi (ODS) rats, a strain not able to synthesize vitamin C." |
| PMID | 11820502 |
| Year | 2002 |
| Species | Rat |
| Journal | J Toxicol Environ Health A |
| RefScore | 1 |
| Source | PArchNLP |
  ||  |  |


---

|  |  |
| --- | --- |
 Reference:3 || Sentence | "As, Ca and Mo had a limited ability to displace Zn from MT while Al, Cr, Fe, Mg, Mn, Tl and V had no effect on Zn binding even at 1.0 mM." |
| PMID | 6695394 |
| Year | 1984 |
| Species | Rat |
| Journal | Toxicol Lett |
| RefScore | 1 |
| Source | PArchNLP |
  ||  |  |


---

|  |  |
| --- | --- |
 Reference:4 || Sentence | "Low levels of iron (LFe) significantly increased the zinc absorption percentage but there was a decrease in high (HFe) and very high iron (VHFe) level groups (p < 0.001)." |
| PMID | 7662501 |
| Year | 1995 |
| Species | Rat |
| Journal | Biol Trace Elem Res |
| RefScore | 1 |
| Source | PArchNLP |
  ||  |  |


---

|  |  |
| --- | --- |
 Reference:5 || Sentence | "Addition of the zinc ligand, histidine, to the water solution decreased the inhibitory effect of the higher dose of iron, resulting in a zinc absorption of 47%." |
| PMID | 3973750 |
| Year | 1985 |
| Species | Human |
| Journal | J Nutr |
| RefScore | 0 |
| Source | PArchNLP |
  ||  |  |


---

|  |  |
| --- | --- |
 Reference:6 || Sentence | Iron supplementation alone increased hemoglobin and ferritin concentrations more than iron and zinc combined. |
| PMID | 16920862 |
| Year | 2006 |
| Species | Human |
| Journal | J Nutr |
| RefScore | 1 |
| Source | PArchNLP |
  |


---

|  |  |
| --- | --- |
